# Theranostics in Renal Cell Carcinoma—A Step Towards New Opportunities or a Dead End—A Systematic Review

**DOI:** 10.3390/ph17121721

**Published:** 2024-12-19

**Authors:** Katarzyna Jóźwik-Plebanek, Marek Saracyn, Maciej Kołodziej, Olga Kamińska, Adam Daniel Durma, Weronika Mądra, Katarzyna Agnieszka Gniadek-Olejniczak, Marek Dedecjus, Jakub Kucharz, Rafał Stec, Grzegorz Kamiński

**Affiliations:** 1 Department of Endocrinology and Radioisotope Therapy, Military Institute of Medicine—National Research Institute, 04-141 Warsaw, Poland; msaracyn@wim.mil.pl (M.S.); mkolodziej@wim.mil.pl (M.K.); adurma@wim.mil.pl (A.D.D.); wmadra@wim.mil.pl (W.M.); gkaminski@wim.mil.pl (G.K.); 2 Department of Nuclear Medicine, Military Institute of Medicine—National Research Institute, 04-141 Warsaw, Poland; okaminska@wim.mil.pl; 3 Neurorehabilitation Clinic, Military Institute of Medicine—National Research Institute, 04-141 Warsaw, Poland; kgniadek-olejniczak@wim.mil.pl; 4 Department of Oncological Endocrinology and Nuclear Medicine, Maria Skłodowska-Curie National Institute of Oncology, 02-781 Warsaw, Poland; 5 Department of Uro-Oncology, Maria Sklodowska-Curie National Research Institute of Oncology, 02-781 Warsaw, Poland; 6 Department of Oncology, Warsaw Medical University, 02-097 Warsaw, Poland

**Keywords:** renal cell carcinoma, positron emission tomography/computed tomography, prostate-specific membrane antigen

## Abstract

**Background:** Renal cell carcinoma is one of the most aggressive urogenital malignancies, with an increasing number of cases worldwide. The majority of cases are diagnosed at an advanced stage, as this form of growth is typically silent. An accurate evaluation of the extent of the disease is crucial for selecting the most appropriate treatment approach. Nuclear medicine imaging is increasingly being applied in oncological diagnostics, prompting ongoing research into renal cell carcinoma markers that could serve as a foundation for theranostic approaches in this disease. Positron emission tomography/computed tomography imaging with prostate-specific membrane antigen (PSMA) ligands has already demonstrated successful utility in diagnosis of other cancers, including prostate cancer and gliomas. Emerging evidence of high sensitivity and specificity in detecting renal cell carcinoma lesions provides a suitable foundation for its application in both the diagnosis and subsequent management of this malignancy. **Methods:** This systematic review synthesizes the current scientific evidence on the molecular imaging of renal cell carcinoma using PSMA ligands, emphasizing the potential future applications of this imaging marker in theranostic approaches. **Results and Conclusions:** Based on a systematic review of the literature, it appears that PET/CT with PSMA ligands has the potential to surpass traditional imaging techniques in diagnostic accuracy while also providing valuable prognostic information.

## 1. Background

Renal cell carcinoma (RCC) accounts for approximately 3% of all adult malignancies, with its incidence having steadily increased over the last 20 years. Currently, around 400,000 new cases of RCC are diagnosed worldwide each year. In 2018, almost 100,000 new cases and almost 40,000 deaths from RCC were recorded in the European Union [1,2,3,4]. RCC is responsible for approximately 90% of renal malignancies, while the remaining 10% comprise cases of lymphoma, sarcoma and metastases from other malignancies located within the kidney. The kidneys are also known to harbor benign lesions, such as adenomas, angiomyolipomas (AML) or oncocytic tumors, which may pose diagnostic challenges. More than half of RCCs are diagnosed incidentally on imaging studies for other causes. The three main histopathologic subtypes of RCC are clear cell carcinoma (ccRCC) (75–80%), papillary carcinoma (pRCC) (15–20%), and chromophobe carcinoma (chrRCC) (5%), of which ccRCC has the worst survival rates, primarily due to its diagnosis occurring at an advanced stage of the disease [5,6].

Imaging anatomical modalities for renal tumors include ultrasound (US), computed tomography (CT), and magnetic resonance imaging (MRI). It should be noted that both CT and MRI may be insufficient to differentiate oncocytic tumors and low-fat AML from RCC lesions. In cases of unclear tumor type or contraindications to CT, MRI with or without contrast is recommended. Percutaneous core-needle biopsies (CNB) of focal renal lesions demonstrate high specificity (98–100%) and sensitivity (86–100%) in diagnosing renal malignancies and are frequently recommended as a reliable method for establishing a definitive diagnosis of RCC [7,8].

It is worth noting that, despite the increased detection of RCC, mainly attributed to the widespread use of diagnostic CT imaging, approximately 25% of patients present with metastatic disease at diagnosis [9,10]. Similarly, about 25% of patients progress to metastatic disease following curative surgery [11,12]. As the incidence of RCC rises, so does the demand for novel diagnostic tools, particularly those enabling precise clinical staging with minimal health risk and guiding the selection of effective therapies. This approach is especially critical in the assessment of advanced disease and in patients previously treated with other therapies. While anatomical imaging methods hold an indispensable role in RCC diagnosis, recent years have brought growing research evidence highlighting the utility of nuclear medicine (NM) techniques in RCC evaluation.

### 1.1. Other Than Prostate-Specific Membrane Antigen (PSMA) Positron Emission Tomography/Computed Tomography (PET/CT) NM Techniques

Conventional imaging modalities, such as contrast-enhanced CT and MRI are the tests of choice in the primary diagnosis and assessment of the local advancement, staging, assessment of treatment response and restaging of ccRCC [13,14].

Classical NM methods, including dynamic scintigraphy with [^99m^Tc]Tc-ethylene-cysteine ([^99m^Tc]Tc-EC), [^99m^Tc]Tc-diethylenetriamine penta-acetic acid ([^99m^Tc]Tc-DTPA), or [^99m^Tc]Tc-mercaptoacetyltriglycine ([^99m^Tc]Tc-MAG3) (all of which are used for the evaluation of renal function), as well as static scintigraphy (optimally using the SPECT/CT technique) with [^99m^Tc]Tc-dimercapto-succinic acid ([^99m^Tc]Tc-DMSA), may play a role in the diagnostic evaluation of focal renal lesions. Neoplastic lesions, abscesses, hematomas, or cysts are observed as areas of absent radiotracer uptake; however, differentiating between these etiologies is not possible using these techniques [15,16,17]. Additionally, static renal scintigraphy with [^99m^Tc]Tc-DMSA can assist in distinguishing between neoplastic tumors and so-called “pseudo-tumors”, which represent anatomical variants of renal shape and structure [15,18]. It is essential to emphasize that, given the availability of alternative diagnostic methods with superior spatial resolution, the role of both dynamic and static scintigraphy in the assessment of focal parenchymal lesions of the kidneys appears to be minimal at present. [^99m^Tc]Tc-methoxy-isobutyl iso-nitrile ([^99m^Tc]Tc-MIBI) scintigraphy may be helpful in ambiguous cases in differentiating RCC and oncocytic tumors (oncocytic tumors, unlike RCC lesions, show high uptake of [^99m^Tc]Tc-MIBI) [19,20]). Scintigraphy with bone tracers, including [^99m^Tc]Tc-methylene diphosphate ([^99m^Tc]Tc-MDP), might be helpful in the detection of (non-specific) bone metastases; however, the most common, i.e., osteolytic, nature of bone metastases from ccRCC remains a limitation of the method [21,22]. None of these methods is included in the current guidelines as a recommended test to assess disease advancement in patients with RCC [7].

[^18^F]-2-fluoro-2-deoxyglucose ([^18^F]FDG) PET/CT, successfully applied in many types of cancers, is not routinely used in the diagnosis and staging of ccRCC. Theoretical limitations of its use result from the physiological elimination of [^18^F]FDG in the kidneys and the variable expression of fructose 1,6-bisphosphatase 1 (GLUT-1) in RCC cells [23,24]. Based on the available literature, it seems that [^18^F]FDG PET/CT has some, but limited, value in the diagnosis and assessment of primary RCC lesions, but it may play a role in the staging of patients with metastatic RCC and with inconclusive results of classic radiological imaging [5,23,24,25,26,27,28,29,30].

The usefulness in RCC diagnostics of other PET tracers, including [^18^F]-fluoride, [^11^C]-acetate, [^68^Ga]Ga-DOTA-TOC, [^124^I]-girentuximab and ([^68^Ga]Ga-fibroblast activation protein inhibitor-04 ([^68^Ga]Ga-FAPI-04), was also assessed in a limited number of studies; however, none of them has found wider application so far [31,32,33,34,35,36,37,38]. The utility of [^18^F]-fluoride PET/CT, similarly to that of scintigraphic bone tracers, is limited due to the predominantly osteolytic nature of RCC bone metastases, which reduces its effectiveness in detecting these lesions [35,37]. The broader application of [^11^C]-acetate PET/CT is constrained primarily by the short half-life of carbon-11, the requirement for an on-site cyclotron, and the high costs associated with the procedure. Studies suggest that [^11^C]-acetate PET/CT may be particularly useful in diagnosing RCC with elevated lipid metabolism, such as higher-grade or specific subtypes, like chrRCC [34]. Additionally, this imaging method may contribute to a deeper understanding of RCC metabolism, potentially aiding in the development of targeted therapies directed at metabolic pathways. However, the limited number of clinical trials conducted to date makes it challenging to fully assess the efficacy of this approach in both diagnosis and treatment planning. [^68^Ga]Ga-DOTA-TOC PET/CT could be particularly beneficial considering the possibility of using long-acting somatostatin analogs or radioligand therapies (RLT) with somatostatin analogs for treatment. However, its utility in RCC is restricted by the generally low expression of somatostatin receptors (SSTRs) in most RCC cases. Notably, certain rare subtypes, such as pRCC or tumors with neuroendocrine components, may exhibit significant SSTR expression, making them potential candidates for this approach [33]. [^124^I]-girentuximab PET/CT shows considerable promise in the diagnostic evaluation of ccRCC due to its high specificity for carbonic anhydrase IX (CAIX), which is highly expressed in the majority of ccRCC cases [38]. Despite its advantages, including exceptional specificity for ccRCC, its current use is limited by high costs and limited availability. In the future, [^124^I]-girentuximab PET/CT may play an integral role in personalized medicine, particularly in the context of targeted therapies and radioimmunotherapy. However, broader application will require further large-scale clinical trials to confirm its utility. [^68^Ga]Ga-FAPI-04 PET/CT demonstrates significant potential, particularly in visualizing the tumor microenvironment in RCC [31,32,36]. Although its current role in RCC diagnostics is constrained by a lack of comprehensive studies and comparative data, [^68^Ga]Ga-FAPI-04 PET/CT could prove valuable in diagnostically challenging cases, monitoring treatment efficacy, and exploring targeted therapies aimed at the tumor microenvironment. Further clinical research is needed to unlock the full potential of this promising technology and expand its clinical applications in the future.

Promising results comparing the usefulness of [^68^Ga]Ga-LNC1007 and [^18^F]FDG PET/CT examination were presented in 2024 by Lin et al. [39]. [^68^Ga]Ga-LNC1007, also known as [^68^Ga]Ga-FAPI-RGD, is a combination of FAPI-02 and cyclic arginine-glycine-aspartate (RGD), a tracer that can detect both fibroblast activation protein (FAP) and αvβ3 integrin. This tracer showed superiority over [^18^F]FDG in detection of primary RCC lesions, (LNC1007 vs. FDG: 13/17 vs. 4/17, *p* = 0.005), and was also able to differentiate lesions in terms of grade of malignancy (assessed with WHO/ISUP grade) and the presence of adverse pathologic features [39]. However, more extensive evaluation is required before this promising tracer can be widely used in the diagnostics of RCC.

### 1.2. PSMA PET/CT Examination

Endothelial cells (ECs) play a crucial role in tumor growth across most cancer types. Genetic and molecular studies have demonstrated that ECs within a variety of solid tumors, including RCCs, exhibit distinct characteristics compared to the typical ECs found in normal arterial vessels. ‘Tip-like’ ECs, a specific subcluster observed in RCC among other cancers, contribute to tumor angiogenesis and immune response inhibition. A high proportion of ‘tip-like’ ECs within a tumor correlates with poor prognosis. PSMA, a transmembrane protein, has been identified as a marker for these ‘tip-like’ ECs [40].

High PSMA expression can be observed in the “tip-like” subcluster of ECs of newly formed blood vessels within cancerous lesions or in the cancer cells themselves. PET/CT with PSMA ligands has been used primarily in the imaging of prostate cancer [41,42]. Due to the overproduction of vascular endothelial growth factor (VEGF) RCC is a highly vascularized tumor [43]. Considering these pathophysiological premises, PET/CT examination with PSMA ligands seems to have a potentially wide application in the diagnosis of RCC, in predicting the effectiveness of different types of treatment, and in assessing the response to treatment.

Increased PSMA expression in ECs of the neo-vasculature of RCC lesions was described for the first time in 2007 by Baccala et al. [44]. These observations were later confirmed by other researchers [45,46,47,48,49,50]. The presence of positive immunohistochemistry (IHC) staining for PSMA is found in 75–100% of ccRCC cases and is the highest among all types of RCC—positive IHC staining was seen only in 30–61% cases of chrRCC and in 0–28% of pRCC [42,45,46,47]. The first PET/CT studies using PSMA ligands ([^68^Ga]Ga-PSMA-11 and [^18^F]-DCFPyL) in the diagnosis of RCC were performed in 2014 [51].

The available literature broadly discusses the following PSMA ligands used in PET/CT imaging:

- [^68^Ga]Ga-PSMA-11 [46,52,53,54,55,56,57,58,59,60],
- [^68^Ga]Ga-PSMA-HBED-CC [61,62,63],
- [^18^F]F-DCFPyL [55,64,65,66],
- [^18^F]F-PSMA-1007 [56,67,68].

**Figure 1:**
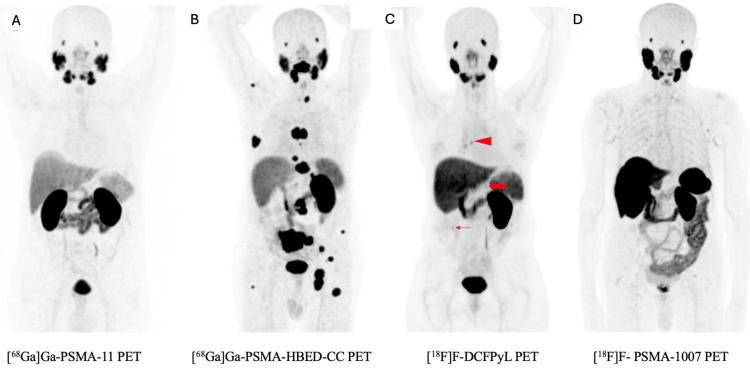
Imaging of RCC with different PSMA-ligands PET. Physiological expression of PSMA is seen in lacrimal and salivary glands, kidneys (proximally convoluted tubules), liver and spleen. (**A**) [^68^Ga]Ga-PSMA-11 PET whole-body maximum intensity projection image [68]. (**B**) [^68^Ga]Ga-PSMA-HBED-CC PET whole-body maximum intensity projection image [61]. (**C**) [^18^F]F-DCFPyL PET whole-body maximum intensity projection image. Multiple foci of abnormal radiotracer uptake (all arrows) [64]. (**D**) [^18^F]F-PSMA-1007 whole-body maximum intensity projection image [68]. All arrows represent multiple foci of abnormal radiotracer uptake.

PSMA ligands can be classified by route of excretion as mainly renal ([^68^Ga]Ga-PSMA-11, [^68^Ga]Ga-PSMA-HBED-CC) or hepatic ([^18^F]F-DCFPyL, [^18^F]F-PSMA-1007), which may be relevant theoretically in the diagnosis of RCC while assessing the primary tumor or recurrence/metastasis located in the kidney or in the liver (Figure 1). The quality of the results may also be influenced by the type of radioisotope used (gallium-68 or fluorine-18). Notably, studies utilizing fluorine-18-labeled ligands are characterized by superior spatial resolution compared to those employing gallium-68.

In recent years, a substantial number of studies have evaluated the efficacy and diagnostic utility of PET/CT imaging with PSMA ligands in ccRCC across various clinical contexts. This systematic review aims to comprehensively analyze and synthesize the available data, focusing on the diagnostic performance of PSMA PET/CT for detecting ccRCC, including both intrarenal lesions and distant metastases. Additionally, the review explores the correlation between PSMA PET/CT findings and histopathological results or [^18^F]FDG PET/CT imaging outcomes. It also examines the impact of PSMA-based diagnostics on modifying patient management strategies and evaluates the relationship between PSMA PET/CT findings and patient prognosis. Furthermore, the latter sections of the article underscore the potential future applications of these imaging tracers in theranostic approaches, emphasizing their promising role in advancing personalized treatment strategies.

## 2. Methods

A systematic literature review was conducted in accordance with Preferred Reporting Items for Systematic Reviews and Meta-Analyses (PRISMA) guidelines. This systematic review was not preregistered in any electronic database. The established review query was: is PSMA PET/CT a useful and/or prognostic tool in imaging diagnostics of ccRCC? According to the PICO framework, the following selections were made: (a) population: patients diagnosed with ccRCC, (b) intervention: diagnostic PSMA PET/CT, (c) comparison: anatomical studies (CT or MRI), histopathological studies, [^18^F]FDG PET/CT, (d) outcomes: correlation between PSMA PET/CT with histopathological results, sensitivity, specificity and detection rate of the PSMA PET/CT study, PSMA uptake indices in PET/CT, impact of PSMA PET/CT on the change in patient management and correlation between the results of PSMA PET/CT examination and patient survival.

Publications available in the Google Scholar and PubMed database concerning the use of PET/CT with PSMA-ligands in ccRCC diagnostics were analyzed. A literature search was performed using a combination of the following key terms: “clear cell renal cell carcinoma PSMA”, “clear cell renal cell carcinoma PET/CT”, “prostate-specific membrane antigen PET/CT”, “renal cell carcinoma PET/CT”, “renal cell carcinoma PSMA”. Available literature was included up to 1 May 2024.

The search was conducted by two researchers (KJP, MS) independently in three stages. In the first stage, the researchers reviewed the databases by key terms and removed duplicate references by PMID and title. The second stage involved screening of article abstracts. Only original papers reporting data on the use of PSMA PET in diagnostics of RCC were included. Researchers excluded articles concerning other than RCC cancers, basic science articles, non-human subjects, guidelines, case reports, editorials, replies, commentary, reviews, and non-English language articles.

**Figure 2:**
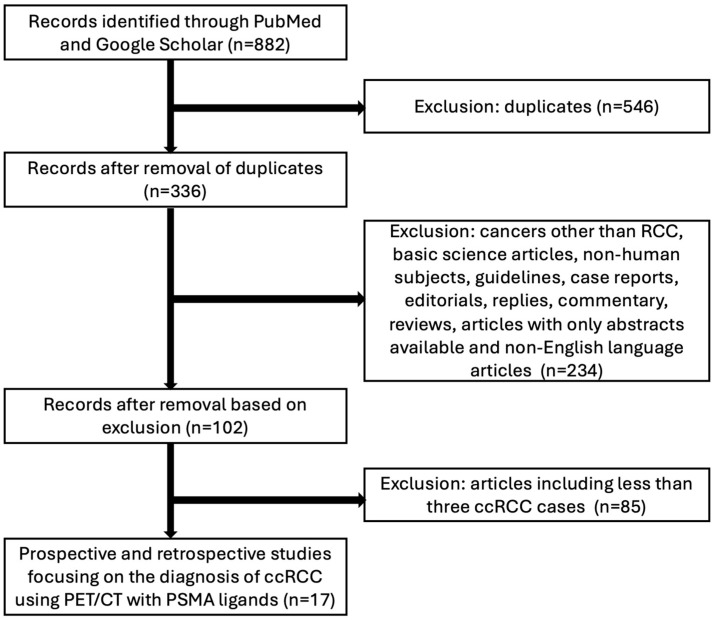
Methodological path. Inclusion and exclusion criteria for systematic review for diagnosis of ccRCC using PSMA-ligands PET/CT.

The third stage involved a full-text analysis of all remaining articles. Data extraction was performed by two independent researchers by reviewing the text, figures, and tables. For each study, the following information was collected: general study details (publication date, country of origin, author details, study aim, study design—prospective or retrospective—and funding); patient demographics (number of RCC and ccRCC cases), baseline clinicopathologic information of patients, PSMA PET/CT indications); PSMA PET/CT technical details (type of PSMA ligand, imaging protocol, patient preparation, injected dose, timing of image acquisition); image analysis (visual, semi-quantitative, quantitative parameters, umber of lesions with uptake on index test); comparative methods and number of lesions on baseline imaging; diagnostic accuracy data (sensitivity, specificity for intrarenal lesions and distant metastases); detection rates (per patient and per lesion); and quantitative measurements, categorized by PSMA ligand type. Studies with less than three patients with ccRCC were excluded from the analysis. The methodological path was presented in the chart below (Figure 2).

Finally, 15 studies regarding use of PSMA PET/CT in the diagnosis of ccRCC were chosen to prepare the final article (Table 1).

**Figure 3:**
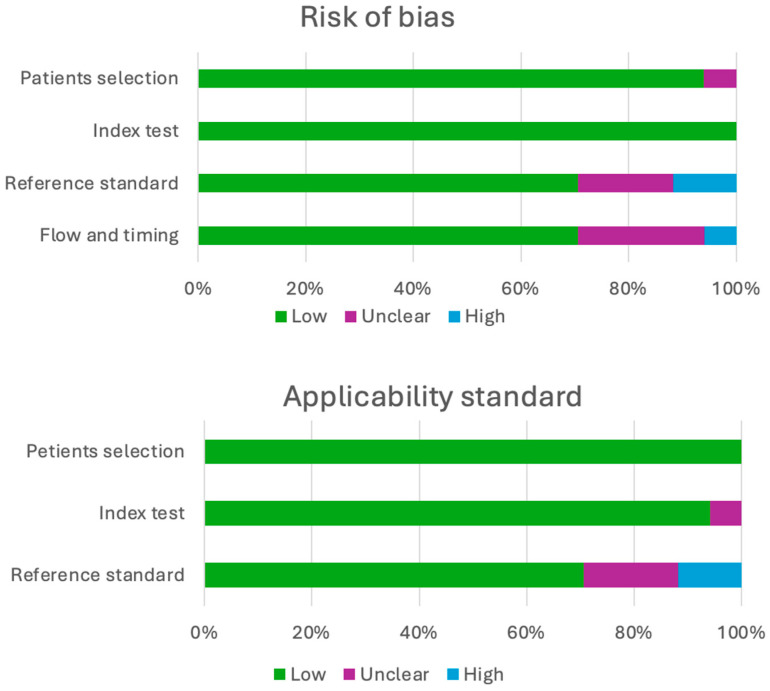
QUADAS-2 results.

The Quality Assessment of Diagnostic Accuracy Studies (QUADAS-2) tool, widely recognized for its effectiveness in evaluating the quality of diagnostic test accuracy studies, was chosen to systematically assess both the risk of bias and the applicability of findings within individual studies (Table 2, Figure 3). Four parameters (patient selection, index test, reference standard, and flow and timing) were considered concerning the risk of bias, and three parameters were evaluated regarding applicability (patient selection, index test, and reference standard). Two reviewers independently assessed the studies’ quality for those included in the systematic review. Any discrepancies among the reviewers regarding quality assessment were resolved by consensus.

## 3. Results

### 3.1. Correlation Between the Histopathological Examination and PSMA PET/CT Results

The majority of the studies show a positive correlation between the uptake of PSMA ligands on PET/CT, assessed by semi-quantitative indices, and the expression of PSMA in tissue samples, assessed by IHC, in both primary and metastatic RCC lesions [52,53,61] (Table 3). Only one study failed to confirm these observations. However, it is important to highlight that the study by Gühne et al. was limited by a small sample size, including only 12 IHC results, compared to the combined 65 ccRCC samples analyzed in the other studies, potentially limiting statistical significance and generalizability [53].

Contrasting to studies evaluating the relationship between PSMA tissue expression and PSMA uptake in PET/CT and studies evaluating the correlation of PSMA PET/CT results with grading, or presence of adverse patho-morphologic features of ccRCC lesions, are still inconclusive [46,53,54,55,67].

The correlation between the WHO/ISUP grade and the results of PSMA PET/CT was evaluated in only two studies. Gao et al. observed a significant correlation between the WHO/ISUP grade of the primary ccRCC lesion and the parameters of [^68^Ga]-PSMA-11 uptake in the preoperative PET/CT study, with area under the curve (AUC) 0.89 (95% CI, 0.81–0.98, *p* < 0.001). The maximal standard uptake value (SUVmax) cut-off point of 16.4 showed a sensitivity of 100% and a specificity of 71% for distinguishing both clinical situations [54]. Once again, contrary to previous studies, the correlation between the WHO/ISUP grade and PET/CT uptake was not confirmed in the study by Gühne et al. All evaluated metastatic lesions of ccRCC, with the exception of bone metastases, were characterized by SUVmax lower than 16.4 assessed in the [^68^Ga]-PSMA-11 PET/CT [53].

Similar discrepancies were observed in studies assessing the correlation between PSMA PET/CT results and the presence of unfavorable pathological features, such as tumor necrosis, sarcomatoid or rhabdoid differentiation [55,62]. The above-mentioned study by Gao et al. also showed a significant correlation between the presence of unfavorable pathological features and the uptake of [^68^Ga]-PSMA-11 in PET/CT, with AUC 0.92 (95% CI, 0.85–0.99, *p* < 0.001). Moreover, the SUVmax cut-off of 18.5 allowed the identification of an adverse pathology with a sensitivity of 94% and a specificity of 87% [54]. Similarly, according to Rhee et al., a particularly high uptake was observed in a ccRCC primary lesion with coexisting sarcomatoid differentiation vs. remaining primary lesions (SUVmax of 28.3 vs. mean SUVmax of the remaining primary lesions 18.0) [62]. In contrast, Udovicich et al. observed no differences in the detection rate of PSMA PET/CT between patients with RCC, with and without coexisting sarcomatoid or rhabdoid differentiation of lesions (90.9% vs. 82.0%; *p* = 0.46), and Tariq et al. also did not note a higher PSMA uptake intensity in metastatic vena cava inferior (IVC) thrombi depending on the presence of adverse patho-morphologic features [55,56].

Attempts were also made to evaluate the correlation between parameters of PSMA uptake in PET/CT and the presence of specific molecular markers that could impact the choice of treatment method and predict response to treatment. As stated in the paper by Gao et al., SUVmax allowed for the differentiation of primary lesions with molecular expression of vascular endothelial growth factor receptor-2 (VEGFR-2), platelet-derived growth factor-β (PDGFR-β) and co-expression of both receptors, which could theoretically be translated into the selection of candidates for treatment with individual tyrosine kinase inhibitors (TKIs) [57]. In the study by Meng et al., the SUVmax of primary RCC lesions in [^68^Ga]Ga-PSMA-11 PET/CT correlated with high hypoxia-inducible factor 2 alpha (HIF-2α) expression, which could potentially be helpful in predicting the response to treatment with HIF-2α antagonists [58].

### 3.2. Diagnostic Accuracy and Uptake Parameters of PSMA PET/CT Studies

Despite the growing interest in PSMA-labeled ligand PET/CT diagnostics in recent years, the number of publications evaluating the diagnostic accuracy of this method in ccRCC/RCC patients is still limited. Additionally, most of these studies are single-centered, involve small groups of patients, and only a few were prospective. However, based on the available literature, it appears that PET/CT imaging with PSMA-labeled ligands has a high diagnostic accuracy both in detecting and assessing the extent of ccRCC. Unfortunately, due to the paucity of data, it is not yet possible to compare the different PSMA ligands.

It is worth mentioning that uptake and presence of pathological lesions on PET/CT can be assessed both visually and, as in other studies, using PET/CT uptake indices. The main variable used in the semi-quantitative analysis of the degree of radiopharmaceutical uptake is SUV (mean, maximum and peak SUV: SUVmean, SUVmax, SUVpeak respectively). Some authors also report the Tumor-to-Background Ratio (TBR) parameter. However, due to significant differences in the selection of “background” areas for TBR assessment, a comparative evaluation of these studies is not possible. 

#### 3.2.1. Sensitivity and Specificity of PSMA PET/CT Studies

Current systematic review of the literature focusing on ccRCC cases identified 12 studies assessing the detection rate of PSMA PET/CT examination—the studies are listed in Table 4 [52,55,56,59,60,61,62,63,64,65,66,67]. The number of patients with ccRCC in individual studies ranged from 4 to 40 and comparative studies were histopathological examinations, CT, MRI or [^18^F]FDG PET/CT studies.

In all the analyzed studies, PSMA PET/CT showed a very high detection rate for ccRCC lesions, mostly exceeding the comparative studies used. The exception to this rule was that of metastatic lesions located in the lungs. After exclusion of lung metastases, “per lesion” analysis was performed, based on which the detection rate for PSMA PET/CT was estimated at 80.5–95.2%, with similar ranges for ligands excreted via the renal and hepatic routes (80.5–95.2 and 88.9–94.7, respectively).

In the retrospective study by Li et al. (focusing on disease limited to 10 distant metastases), the sensitivity, specificity, and diagnostic accuracy of [^68^Ga]Ga-PSMA-11 PET/CT (a tracer excreted via the renal route) for detecting ccRCC, in comparison to conventional radiological imaging (CT and/or MRI), were reported as 93.6%, 97.9%, and 96.6%, respectively [52]. The smallest lesion dimension detected by [^68^Ga]Ga-PSMA-11 PET/CT, as noted by the authors, was 8 mm [52]. Raveenthiran et al., in their retrospective case series involving 28 patients with ccRCC, demonstrated a sensitivity of 80.5% for [^68^Ga]Ga-PSMA-11 PET/CT on a “per lesion” analysis, encompassing both staging and restaging contexts [59]. In a prospective study by Aggarwal et al., which was not included in prior meta-analyses, [^68^Ga]Ga-PSMA-11 PET/CT identified more tumor thrombi and ccRCC lesions within bone marrow compared to CT [60]. Additionally, the prospective study by Rhee et al., which included 10 patients with newly diagnosed RCC (8 with ccRCC, 1 with pRCC, and 1 with unclassified RCC), identified a total of 86 tumor lesions. The sensitivity of PET/CT using another kidney-excreted tracer, [^68^Ga]Ga-PSMA-HBED-CC, was 92.11% (CI 0.78–0.98), significantly higher than the sensitivity of CT, which was only 68.6% (CI 0.51–0.83). The positive predictive value (PPV) of PSMA PET/CT was 97.22% (CI 0.84–1.00), compared to the PPV of CT, which was 80% (CI 0.61–0.92). Notably, the smallest lymph node detected by PET/CT was 6 mm [62].

Regarding tracers primarily excreted via the hepatic route, a prospective study by Meyer et al. evaluated 14 patients with presumed oligometastatic ccRCC (up to three metastatic lesions identified on conventional imaging) using [^18^F]F-DCFPyL PET/CT for primary staging. The detection rate of PSMA PET/CT was 92.8% on a “per patient” analysis and 88.9% on a “per lesion” analysis, with CT and/or MRI serving as reference imaging modalities. Importantly, PSMA PET/CT identified more metastatic lesions than conventional imaging, detecting a total of 29 metastatic lesions compared to 21 visible on CT/MRI, including 12 additional lesions that were undetected by conventional methods [64]. In a retrospective study by Liu et al., which included 15 patients with ccRCC, [^18^F]F-DCFPyL PET/CT demonstrated a sensitivity of 100% for detecting both local recurrence and distant metastases in both per patient and per lesion analyses [65].

A notable issue identified in several analyzed studies is the relatively low sensitivity of PSMA PET/CT for detecting metastatic lesions in the lungs. According to Sawicki et al., [^68^Ga]Ga-PSMA-HBED-CC PET/CT successfully detected only 50% of ccRCC metastatic lung lesions. The dimensions of lung lesions visible on PET/CT were 1.4 ± 0.4 cm, while PET/CT-negative lesions measured 0.7 ± 0.2 cm [63]. Similarly, in the study by Li et al., [^68^Ga]Ga-PSMA-11 PET/CT demonstrated high sensitivity (>90%) for lesions in most anatomical locations; however, its sensitivity for lung lesions was notably lower, at 71.43%, while specificity remained above 90% [52]. Furthermore, in a prospective study conducted by Rhee et al., the smallest lung lesion with detectable tracer accumulation measured 9.6 mm [62].

The reduced sensitivity of PET/CT for pulmonary lesions is likely attributable to respiratory motion during PET acquisition, combined with the inherently lower spatial resolution of PET imaging. Given these limitations, CT remains the preferred modality for evaluating lung metastases in RCC patients.

The detection rate/diagnostic accuracy of PSMA PET/CT studies in both “per patient” and “per lesion” analysis for RCC has been assessed in two large meta-analyses in recent years [69,70].

Rizzo et al. in their meta-analysis published in 2023, which included 14 predominantly retrospective studies (total number of patients 397 (range 8–66), including 331 patients with ccRCC), evaluated the diagnostic accuracy of PSMA PET/CT in RCC diagnostics, regardless of the type of PSMA ligand used, both at the stage of primary disease advancement and during restaging of ccRCC in patients with suspected metastatic disease [70]. The detection rate of PET/CT was excellent, ranging from 84% to 100% in the per patient analysis and from 80.5% to 100% in the per lesion analysis. Comparative studies referenced by the authors include conventional radiologic examinations, such as CT or MRI and/or [^18^F]FDG PET/CT. The average (mean/median) SUVmax for primary lesions and for metastatic lesions was 6.9–25.9 and 2.7–19.5, respectively. In most articles, PSMA PET/CT detected more lesions than CT, MRI, or [^18^F]FDG, particularly bone metastases. The described locations of metastatic lesions included lymph nodes, adrenal glands, lungs, liver, contralateral kidney, bones, brain and other uncommon locations, such as the pancreas. In only one article did PSMA PET/CT show a lower detection rate than CT (83.6% vs. 93.4%) [55].

Furthermore, according to the meta-analysis by Singhal et al. from 2024, including 11 papers published between 2015 and 2023, PSMA PET/CT scans had a pooled sensitivity for ccRCC of 94.7% (95% CI: 88–98.3%) in the “per patient” analysis and a pooled sensitivity of 91.0% (95% CI: 86.4–94.4) in the “per lesion” analysis [71].

#### 3.2.2. Assessment of Kidney Lesions

Lesions located in the kidneys, including primary tumors, local recurrences and kidney metastases, were assessed using PET/CT with PSMA ligands in nine studies. All these studies as well as evaluated parameters are listed in Table 5.

Six studies evaluated the sensitivity of PSMA PET/CT, defined as visually detectable uptake in RCC lesions within the kidneys [51,52,59,61,64,67]. The sensitivity for PSMA ligands excreted via the renal route ranged from 62% to 100%, which was lower compared to ligands predominantly excreted through the hepatic route, where sensitivity ranged from 80% to 100%. Sensitivity specific to ccRCC kidney lesions was assessed in only four studies. Notably, in the study by Raveenthiran et al., at the same time, uptake was observed in as many as 40% of benign lesions, suggesting that qualitative analysis may have limited utility in the differential diagnosis of focal renal lesions.

In the literature, the semi-quantitative PSMA PET/CT parameters of both RCC and ccRCC lesions located in the kidneys seems to be higher for radiopharmaceuticals mainly excreted by the kidneys than for those excreted via the liver route. Based on available data, SUVmax for RCC lesions ranged from 1.7 to 114.6 for radiopharmaceuticals mainly excreted by the kidneys and from 3.2 to 15.8 for those excreted via the liver route (Table 5).

In the study by Li et al., using [^68^Ga]Ga-PSMA-11 PET/CT, renal lesions demonstrated a higher SUVmax compared to metastatic lesions. The median SUVmax for renal lesions was 18.0, whereas the median SUVmax for metastatic lesions ranged from 7.4 to 9.6 [52]. Gasparro et al., employing [^68^Ga]Ga-PSMA-HBED-CC PET/CT (another tracer excreted via the renal route), reported a mean SUVmax of 34.1 for primary renal lesions. Among metastatic sites, the highest uptake was observed in bone and liver lesions, with mean SUVmax values of 28.6 and 29.8, respectively—both lower than the SUVmax for kidney lesions [61]. Similarly, in the prospective study by Rhee et al., which involved 10 patients with newly diagnosed RCC, the average SUVmax for all 10 primary renal lesions was 18 (range 3.7–36.5), comparable to metastatic lesions, which had an average SUVmax of 19.5 (range 1.5–48.0) [62].

For tracers primarily excreted via the hepatic route, the prospective study by Meyer et al. evaluated [^18^F]F-DCFPyL PET/CT for primary staging. The median SUVmax for primary lesions was 9.6 (range 7.3–15.8), while metastatic lesions had a median SUVmax of 2.7 (range 0.9–38.5) [64]. Additionally, in the retrospective study by Liu et al., [^18^F]F-DCFPyL PET/CT showed an advantage over [^18^F]FDG PET/CT by accurately differentiating postoperative renal changes from local recurrence in all six patients [65].

#### 3.2.3. Assessment of Metastatic Lesions

The uptake parameters for ccRCC metastases and the sensitivity of PSMA PET/CT studies in relation to other imaging modalities and histopathological examinations were assessed in 11 studies, most of which were retrospective (Table 6 and Table 7) [52,53,59,61,64,65].

Considering the type of PSMA ligand used (divided in terms of way of excretion) and the location of extrarenal metastatic lesions, uptake parameters were comparable for both types of radiopharmaceuticals, with the SUVmax ranging from 1.2 to 66.6 and 0.9 to 38.5 for PSMA ligands excreted via hepatic and kidney route, respectively.

In nine studies, semi-quantitative uptake parameters and detection rates for RCC metastases in individual organs were available. Detailed data are shown in Table 7. Unfortunately, no studies have reported independent uptake parameters in metastatic lesions of ccRCC cases. Of note, none of the locations of RCC metastases can be highlighted as having the significantly highest uptake values in all studies.

### 3.3. Impact of PSMA PET/CT Examination on Treatment Management

The reviewed literature demonstrates a significant impact of PSMA PET/CT on alterations in patient management at both the staging and restaging phases. It can be concluded that performing PSMA ligand PET/CT significantly influences clinical decision-making in 13–43.8% of patients compared to treatment plans based solely on conventional imaging methods. The primary reason for this impact is the identification of new metastatic lesions that were undetectable in conventional imaging, leading to a reclassification from locoregionally confined or oligometastatic disease to a diagnosis of metastatic disease [52,59,60,61,62]. None of the tracers, as well as none of the tracer types (excreted via the renal or hepatic route), was definitely better than the others, although the highest modifying impact was revealed by Raveenthiran et al. using [^68^Ga]Ga-PSMA-11 [59]. The authors proved that the use of [^68^Ga]Ga-PSMA-11 PET/CT directly influenced treatment decisions in 43.8% of patients at primary staging and in 40.9% at restaging [59].

According to Li et al. ([^68^Ga]Ga-PSMA-11), Rhee et al. ([^68^Ga]Ga-PSMA-HBED-CC), and Meyer et al. ([^18^F]F-DCFPyL), performing PSMA PET/CT at the time of primary staging of the disease led to a change in management in 12.9%, 20%, and 21.4% of patients, respectively [52,62,64]. A change in patients’ management during restaging of the ccRCC after PSMA PET/CT was also observed in 49% of patients in the study by Udovicich et al. [55]. In this study, the authors used two different tracers ([^68^Ga]Ga-PSMA-11 or [^18^F]F-DCFPyL) interchangeably, depending on their availability, and the results do not include the division into both types.

Besides commencement of systemic therapy in patients with newly diagnosed metastatic disease, performing PSMA PET/CT also resulted in the planning of radiotherapy (stereotactic ablative body radiotherapy) or surgical treatment in patients with single metastatic lesions [61].

### 3.4. Impact of PSMA Expression on Patient Survival

In only three studies was a correlation between PSMA PET/CT results and patient survival assessed. Notably, all three studies demonstrated that the findings from PSMA PET/CT examinations correlated with patient prognosis [50,60,61].

Higher PSMA expression, determined in IHC stained specimens, was significantly correlated not only with higher grade and pT stage of ccRCC, but also with the presence of distant metastases and shorter overall survival (OS) at the end of a 10-year follow-up, as observed by Spatz et al. [50]. Prospective studies of RCC patients also noted differences in patient survival based on PSMA PET/CT results. According to Gasparro et al., the median survival of patients was significantly higher in RCC patients (of whom 84.6% were diagnosed with ccRCC) with a negative (48 months) than with a positive PSMA PET/CT result (24 months) (*p* = 0.001, hazard ratio (HR) 3.6, 95% CI 1.36–9.79) [61]. A cut-off value of SUVmax ≥ 7.4 differentiated patients with poor prognosis (mean survival time 24 months) from other patients (mean survival time 33 months) (*p* = 0.01, HR 0.28, 95% CI 0.10–0.77) [61]. Analyzing only patients with ccRCC, the median survival in the group with SUVmax ≥ 7.4 was 24 months; while in the group with SUVmax < 7.4, this was 51.5 months (*p* = 0.03, HR 0.29, 95% CI 0.10–0.83). In the same study, the authors analyzed the relationship between survival of ccRCC patients and PSMA expression in IHC, and observed different median OS of 37.5, 24, and 34 months in groups with low or no expression, moderate expression, and high expression of PSMA, respectively, although the results did not reach statistical significance (*p* = 0.20) [61]. In the study by Aggarwal et al. high number of PSMA-positive lesions, high baseline PSMA-derived Tumor Volume (PSMA-TV) and Total Lesion PSMA (TL-PSMA) were all significantly correlated with OS of ccRCC patients with HR 6.7, 8.9, 8.9 [60].

### 3.5. PSMA PET/CT Versus [^18^F]FDG PET/CT Imaging

The comparison of two PET tracers ([^18^F]FDG and PSMA ligands) has been the subject of research by several authors (Table 8).

As reported by Udovicich et al., a subgroup of 40 RCC patients, most with cancerous lesions, showed uptake on both [^18^F]FDG PET/CT and PSMA PET/CT, but in almost all patients the uptake values were lower on [^18^F]FDG PET/CT [55]. In 5% of patients, the lesions showed uptake only in [^18^F]FDG PET/CT (PSMA PET/CT negative), which the authors attributed to the heterogeneity and possible dedifferentiation of the tumor lesions. As stated by Liu et al., PSMA PET/CT detected more metastatic ccRCC lesions within soft tissues (14/14 vs. 10/14, *p* = 0.12) than [^18^F]FDG PET/CT; moreover, it showed a higher detection rate in detecting local recurrence of RCC and bone metastases [65]. In the study by Tariq et al., in which each RCC patient (91% ccRCC) underwent both [^18^F]FDG PET/CT and PSMA PET/CT for primary tumor evaluation, uptake of both tracers was seen in 40%, PSMA uptake alone in 20%, and [^18^F]FDG uptake alone in 40% of lesions. For metastatic lesions, concordance between the two PET/CT studies was found in 55% of patients with positive results, 27% with negative results, and 18% with PSMA uptake alone [67]. In the prospective study by Aggarwal et al., including 24 ccRCC patients, [^68^Ga]Ga-PSMA-11 PET/CT showed more ccRCC lesions than [^18^F]FDG PET/CT, especially in bone and lung and the lesions had significantly higher parameters of tracer uptake (median SUVmax 6.9 vs. 5.2, *p* < 0.001; TBR 5.7 vs. 3.8, *p* < 0.001) [60]. Noteworthy, the median SUVmax of renal lesions (primary tumor) was higher for [^68^Ga]Ga-PSMA-11 PET/CT (16.2 vs. 5.5, *p* = 0.002) despite lower median TBR than for [^18^F]FDG PET/CT (0.7 vs. 3.9, *p* < 0.001), probably influenced by the route of excretion of [^68^Ga]Ga-PSMA-11. All except liver metastases had higher SUVmax on [^68^Ga]Ga-PSMA-11 than on [^18^F]FDG PET/CT [60].

Although most lesions are detectable with both types of tracers (PSMA and [^18^F]FDG), the authors believe that, at least until more extensive scientific evidence is available, these imaging modalities should not be regarded as interchangeable but rather as complementary. This approach is analogous to the molecular imaging of SSTR and [^18^F]FDG PET/CT in neuroendocrine tumors.

Given previous study findings highlighting differences in patient prognosis based on separate [¹⁸F]FDG PET/CT and PSMA PET/CT results, prospective research evaluating the prognostic impact and response to systemic treatment using both imaging modalities simultaneously would be highly valuable. However, such studies have not yet been published. Considering the results of comparative studies involving both types of radiotracers (PSMA and [¹⁸F]FDG) and the observation that certain RCC lesions are detectable only on [¹⁸F]FDG scans, an optimal approach from the patient’s perspective would involve performing both examinations prior to determining the treatment method and type. This is particularly crucial in cases where anatomical imaging reveals lesions that are not visible on PSMA PET/CT, as seen in the treatment of neuroendocrine tumors.

### 3.6. PSMA Ligand Therapy Perspectives

In recent years, the efficacy of RLT using PSMA ligands ([^177^Lu]Lu-PSMA-617, [^177^Lu]Lu-PSMA-I&T, and [^225^Ac]Ac-PSMA-617) in hormone-refractory metastatic prostate cancer (mCRPC) has been reported. The undeniable advantage of this therapy is its good tolerability and low number of side effects.

Among the available PSMA ligands, the majority of safety data stems from clinical trials on prostate cancer treatment using [^177^Lu]Lu-PSMA-617, including the phase III VISION trial and phase II studies such as TheraP and RESIST-PC [71,72,73]. RLT with [^177^Lu]Lu-PSMA-617 is considered relatively safe, with a low incidence of serious adverse events. The most common adverse events include fatigue (reported in 24% of patients in the VISION trial), dry mouth (xerostomia) (38.8%), nausea, and myelosuppression.

In the VISION trial, anemia was the most frequent manifestation of myelosuppression, affecting 31.8% of patients, with grade 3–4 anemia observed in 12.9%. Gastrointestinal toxicities included nausea (35.3%), constipation (20.2%), vomiting (18.9%), diarrhea (18.9%), and abdominal pain (6.0%). Myelosuppression, encompassing anemia, thrombocytopenia, and leukopenia, was the most common reason for dose reduction, interruption, or discontinuation of RLT [71].

The VISION trial demonstrated significant improvements in progression-free survival (PFS) and OS compared to standard care, with a median OS of approximately 15 months in the RLT group versus 11.3 months in the control group [71].

Treatment outcomes are influenced by several factors. Higher PSMA expression, as determined by PSMA PET/CT imaging prior to therapy, correlates with better efficacy. Conversely, the presence of PSMA-negative lesions, which are non-responsive to [^177^Lu]Lu-PSMA-617, can reduce treatment effectiveness. Patients with a lower disease burden, better overall performance status, and limited prior systemic therapy are more likely to benefit from RLT. Although individualized dosimetry could theoretically enhance treatment outcomes by optimizing radiation delivery and minimizing toxicity to healthy tissues, there is currently insufficient clinical trial data to broadly implement this approach.

Given the proven overexpression of PSMA in ECs or cancer cells of other solid tumors, attempts are being made to apply PSMA ligand-based RLT to other clinical entities such as gliomas, salivary gland carcinomas, papillary thyroid cancer and hepatocellular carcinoma [74,75,76,77].

It appears that at least some ccRCC patients with high PSMA expression may benefit from RLT. The first report of RLT in RCC came from Germany in 2023 [78]. Zhang et al. described the case of a 71-year-old patient with metastatic RCC (pleural and lymph node metastases) who received one cycle of [^177^Lu]Lu-PSMA I&T (5.9 GBq). Unfortunately, the authors observed rapid washout of the radiopharmaceutical from the cancerous lesions—uptake in metastatic lesions was visible only on the scintigraphy scans performed 2.5 h after administration of the radiopharmaceutical, but not in subsequent imaging studies (at 19.5 h, 43.5 h, and 68 h) [78]. It appears that the rapid washout of PSMA ligands from ccRCC lesions may result from PSMA expression in the ECs of the neo-vasculature rather than directly in cancer cells and may be a factor that hinders the treatment of RCC with PSMA-labeled ligands due to the lower radiation doses absorbed by the cancer cells.

Currently, the first prospective clinical trial (National Clinical Trial number (NCT) at Clinicaltrals.gov: NCT06059014) titled *Phase I/II Study Evaluating PSMA Targeted Radionuclide Therapy in Adult Patients with Metastatic Clear Cell Renal Cancer* (PRadR) has begun enrollment and is expected to provide data on the safety and efficacy of RLT with [^177^Lu]Lu-PSMA-1 in ccRCC. Recruitment is expected to be completed by April 2026, with a total of 48 patients expected to be enrolled.

Similarly to [^177^Lu]Lu-PSMA therapy for prostate cancer, attempts are also being made to modify the PSMA molecule in order to increase the uptake of the radiotracer by the neoplastic lesion. One way to modify the PSMA molecule is by combining it with Evans blue (EB), which prolongs its binding time with blood proteins. In patients with mCRPC, the use of [^177^Lu]Lu-EB-PSMA-617 was associated with a 3-fold increase in accumulated activity by metastatic lesions but also with simultaneous 6-fold increase in absorbed doses in red marrow and kidneys—compared to therapy with [^177^Lu]Lu-PSMA-617 [79]. In the study of Zang et al. 28 patients with prostate cancer received up to three cycles of [^177^Lu]Lu-EB-PSMA-617 treatment with different doses in each of three groups (1.2 GBq, 2.1 GBq and 3.5 GBq, respectively). The treatment was highly effective but associated with a high degree of myelotoxicity (25% grade 4 thrombocytopenia in the 3.5GBq group) [80]. It seems that in the case of treatment of ccRCC with [^177^Lu]Lu-EB-PSMA-617 the ratio of the dose absorbed by the neoplastic lesions to the toxic doses received by the critical organs will be even more unfavorable than in the case of prostate cancer, due to the different location of PSMA (on the surface of the neovascular endothelium in the cases of ccRCC and on the surface of the cancer cell in the case of prostate cancer), and that this direction of research on the use of PSMA ligands in the treatment of renal cancer will also turn out to be a dead end. The only clinical trial (40 patients) (NCT05170555) testing [^177^Lu]Lu-EB-PSMA-617 treatment in patients with ccRCC was completed in July 2023, but the results have not been published till now.

### 3.7. Treatment with Other Experimental Radiotracers

CAIX is a transmembrane glycoprotein that is expressed in cells of ccRCC. Radiolabeled with [^124^I] or [^89^Zr] monoclonal antibodies anti-CAIX (girentuximab) were examined as a potential radiotracer in ccRCC PET/CT diagnostics. In a study conducted by Divgi and et al., [^124^I]I-girentuximab demonstrated satisfactory accuracy in the identification of ccRCC lesions among other renal masses, with a sensitivity of 86.2% (95% CI 75.3–97.1%) and a specificity of 85.9% (95% CI 69.4–99.9%) [81]. In the *Zirconium in Renal Cancer Oncology* trial, [^89^Zr]Zr-DFO-girentuximab demonstrated a comparable high sensitivity and specificity in discriminating ccRCC from non-ccRCC primary tumors (85.5% and 87%, respectively) [82]. In the initial phase of the Japanese *Zirdac-JP* study [^89^Zr]Zr-DFO-girentuximab demonstrated promising outcomes with respect to its safety profile, biodistribution, and dosimetry, indicating its potential as a novel PET/CT tracer for ccRCC [83]. It is also noteworthy that the SPECT/CT technique, utilizing [^111^In]In-girentuximab, has been evaluated as a potentially valuable and efficacious approach for the monitoring of patients following cryoablation for ccRCC [84]. It is unfortunate that the initial applications of [^177^Lu]Lu-girentuximab in the treatment of ccRCC have yielded unfavorable outcomes, primarily due to the high incidence of myelotoxicity.

An innovative approach to the treatment of ccRCC is the combination of subtherapeutic doses of radiation delivered by [^177^Lu]Lu-DOTA-girentuximab and Programmed Cell Death-1/Cytotoxic T-lymphocyte antigen 4 (PD-1/CTLA-4) immunotherapy. In animal models, this combined treatment demonstrated high therapeutic efficacy, which may provide further directions for the development of research with anti-CAIX radiolabeled antibodies. Currently, two clinical studies (NCT05663710, NCT05239533) are underway to assess the safety and efficacy of a combination therapy based on girentuximab in humans.

An area of theranostics in renal cancer that remains unexplored is diagnostics and treatment with Programmed Cell Death-1 ligands (PD-1L). The initial investigations into the diagnostic applications of PD-1L were conducted in other neoplasms [85]. It appears that NM imaging may prove valuable, at least in terms of predicting the response to PD-1/PD-1L-based therapies, in the context of ccRCC.

## 4. Conclusions

The key conclusions from the conducted systematic review of the literature on PSMA PET/CT examination in ccRCC regarding its diagnostic utility and prognostic value are as follows:

- As found in most studies, it seems that the uptake in PSMA PET/CT reflects tissue expression of PSMA. However, conflicting results preclude confirming a definitive relationship between the intensity of PSMA PET/CT uptake, the grade of ccRCC lesions, and the presence of unfavorable pathological features. Considering the findings of the correlation between PSMA expression assessed IHC and patient survival, definitively establishing a relationship between PSMA expression and uptake in PET/CT studies would provide a unique, non-invasive tool as one of the key elements in assessing patient risk.
- PSMA PET/CT demonstrates very high sensitivity in detecting ccRCC lesions, including both intrarenal and metastatic sites, frequently surpassing conventional imaging methods. A notable exception is intrapulmonary metastatic lesions, for which CT imaging appears to outperform PSMA PET/CT.
- For intrarenal ccRCC lesions, sensitivity appears lower when using PSMA ligands excreted via the renal route. However, these ligands often show higher semi-quantitative uptake parameters compared to ligands primarily excreted via the hepatic route.
- PSMA PET/CT significantly influences treatment strategies, with 13–43.8% of patients experiencing changes in their management plans compared to those based solely on conventional imaging
- While only limited studies, including one prospective study, are available, higher PSMA PET/CT uptake appears to correlate with a poorer prognosis for ccRCC patients.
- Due to the limited number of published studies, direct comparisons of individual PSMA ligands regarding specificity and diagnostic utility are not yet feasible.
- Initial attempts to use PSMA ligands for radioligand therapy in ccRCC have been disappointing. Rapid radiotracer washout from neoplastic lesions limits the energy delivered to pathological tissues, rendering this approach insufficient at the current stage.

It appears that the use of PET/CT with PSMA ligands may offer superior diagnostic performance compared to conventional imaging methods, while also providing valuable prognostic information. However, no single PSMA ligand has yet been demonstrated to outperform others in terms of sensitivity or specificity for detecting RCC lesions. Additionally, there is no evidence to suggest that individual PSMA ligands vary in their affinity for different locations, subtypes of ccRCC, or tumor grades. In the authors’ view, the primary limitation at this stage is the lack of prospective studies involving large patient cohorts that compare both types of markers (those excreted via the renal and hepatic routes) in the evaluation of renal lesions, including local recurrences, as well as metastatic liver lesions. Large-scale studies comparing individual PSMA ligands could also provide important information on differences in sensitivity of PET/CT studies and their impact on the prognosis of patients, depending on the radioisotope used (based on the physical properties of radioisotopes, we would expect higher spatial resolution and therefore sensitivity in studies using fluorine-18 compared to studies with gallium-68). However, there are no such comparative studies in this area so far.

Another important area for further investigation is whether imaging with PSMA ligands in patients with ccRCC can serve as a foundation for selecting the most appropriate therapy, thus enabling personalized treatment. This approach has become a central goal in both nuclear medicine and oncology in recent years. However, the published case raises concerns about the efficacy and safety of the therapy. In the light of experience gained with RLT in the treatment of other tumors, it is imperative to modify the PSMA molecule in order to prolong the retention of the PSMA-radioisotope complex in cancer lesions. An illustrative example is Evans blue (such attempts have thus far been limited to patients with prostate cancer) or PSMA cyclization (analogous to attempts made with cyclic fibroblast activation protein inhibitor (FAPI) in animal models).

Given the multitude of issues related to the diagnosis and therapy of ccRCC using PSMA ligands, further research is encouraged. Additionally, it should be mentioned that the principles of theranostics have the potential to be applied to other receptors or surface antigens specific to renal cancer cells, which, according to the authors of this review, could indicate a promising step towards new opportunities in theranostics for renal cancer patients.

## Figures and Tables

**Table 1:** Basic characteristics of the studies included in the systematic review.

Authors [Ref.]	Number of CcRCC Patients Included	Year	Country	Tracer	Study Design	Comparative Imaging/Tests
Golan et al. [46]	18	2021	Israel	[^68^Ga]Ga-PSMA-11	prospective	histopathology
Li et al. [52]	40	2022	China	[^68^Ga]Ga-PSMA-11	retrospective	CT and/or MRI, histopathology
Ghune et al. [53]	9	2021	Germany	[^68^Ga]Ga-PSMA-11	prospective	histopathology
Gao et al. [54]	36	2020	China	[^68^Ga]Ga-PSMA-11	retrospective	histopathology
Udovicich et al. [55]	54	2022	Australia	[^68^Ga]Ga-PSMA-11, [^18^F]F-DCFPyL	retrospective	CT, [^18^F]FDG PET/CT
Tariq et al. [56]	14	2022	Australia	[^18^F]F-PSMA-1007, [^68^Ga]Ga-PSMA-11	retrospective	MRI
Gao et al. [57]	34	2022	China	[^68^Ga]Ga-PSMA-11	retrospective	histopathology
Meng et al. [58]	40	2022	China	[^68^Ga]Ga-PSMA-11	retrospective	histopathology
Raveenthiran et al. [59]	28	2019	Australia	[^68^Ga]Ga-PSMA-11	retrospective	CT
Aggarwal et al. [60]	27	2024	India	[^68^Ga]Ga-PSMA-11	prospective	CT, [^18^F]FDG PET/CT
Gasparo et al. [61]	22	2023	Italy	[^68^Ga]Ga-PSMA-HBED-CC	prospective	CT and/or MRI, histopathology
Rhee et al. [62]	8	2016	Australia	[^68^Ga]Ga-PSMA-HBED-CC	prospective	CT, histopathology
Sawicki et al. [63]	4	2017	Germany	[^68^Ga]Ga-PSMA-HBED-CC	retrospective	CT, histopathology
Meyer et al. [64]	14	2019	USA	[^18^F]F-DCFPyL	prospective	CT and/or MRI
Liu et al. [65]	15	2020	China	[^18^F]F-DCFPyL	retrospective	[^18^F]FDG PET/CT
Rowe et al. [66]	5	2015	USA	[^18^F]F-DCFPyL	retrospective	CT and/or MRI
Tariq et al. [67]	10	2022	Australia	[^18^F]F-PSMA-1007	retrospective	[^18^F]FDG PET/CT

ccRCC—clear cell renal cell carcinoma, CT—computed tomography, MRI—magnetic resonance imaging, PET/CT—positron emission tomography/computed tomography, PSMA—prostate-specific membrane antigen.

**Table 2:** QUADAS-2 results.

Authors [Ref.]	Risk of Bias				Applicability Standard		
	Patients Selection	Index Test	Reference Standard	Flow and Timng	Patent Selection	Index Test	Reference Standard
Golan et al. [46]	Low	Low	Low	Low	Low	Low	Low
Li et al. [52]	Low	Low	Low	Low	Low	Low	Low
Ghune et al. [53]	Low	Low	Low	Unclear	Low	Low	Low
Gao et al. [54]	Low	Low	Low	Unclear	Low	Low	Low
Udovicich et al. [55]	Unclear	Low	High	Low	Low	Unclear	High
Tariq et al. [56]	Low	Low	Low	Low	Low	Low	Low
Gao et al. [57]	Low	Low	Low	Low	Low	Low	Low
Meng et al. [58]	Low	Low	Low	Low	Low	Low	Low
Raveenthiran et al. [59]	Low	Low	Low	Unclear	Low	Low	Unclear
Aggarwal et al. [60]	Low	Low	Unclear	Unclear	Low	Low	Unclear
Gasparo et al. [61]	Low	Low	Unclear	Low	Low	Low	Unclear
Rhee et al. [62]	Low	Low	Low	Low	Low	Low	Low
Sawicki et al. [63]	Low	Low	Low	Low	Low	Low	Low
Meyer et al. [64]	Low	Low	High	Low	Low	Low	High
Liu et al. [65]	Low	Low	Unclear	Low	Low	Low	Low
Rowe et al. [66]	Low	Low	Low	High	Low	Low	Low
Tariq et al. [67]	Low	Low	Low	Low	Low	Low	Low

**Table 3:** Relationship between the ccRCC histopathological examination and PSMA PET/CT results.

Authors [Ref.]	Number of ccRCC Patient Included	Tracer	PSMA PET/CT and Histopathological Evaluated Parameters
Li et al. [52]	40	[^68^Ga]Ga-PSMA-11	correlation of SUVmax with tissue expression of PSMA (*p* = 0.035)
Ghune et al. [53]	9	[^68^Ga]Ga-PSMA-11	correlation between: - SUVmax and intensity of tissue PSMA expression (*r* = 0.245, *p* = 0.467) - SUVmax and lesion grade (*r* = −0.215, *p* = 0.525) - no correlation between WHO/ISUP grade and PET/CT uptake
Gao et al. [54]	36	[^68^Ga]Ga-PSMA-11	correlation between: - WHO/ISUP grade (3–4 vs. 1–2) and PSMA PET/CT uptake (*p* < 0.001) - presence of unfavorable pathological features and PSMA PET/CT uptake (*p* < 0.001)
Udovicich et al. [55]	54	[^68^Ga]Ga-PSMA-11 [^18^F]F-DCFPyL	no differences between RCC with and without coexisting sarcomatoid/rhabdoid differentiation of lesions
Tariq et al. [56]	14	[^18^F]F-PSMA-1007	no correlation between PSMA PET/CT uptake and the presence of adverse pathomorphologic features in metastatic IVC thrombi
Gasparo et al. [61]	22	[^68^Ga]Ga-PSMA-HBED-CC	correlation of SUVmax with tissue expression of PSMA (*r* = 0.33; *p* = 0.01)
Rhee et al. [62]	8	[^68^Ga]Ga-PSMA-HBED-CC	high PSMA PET/CT uptake in ccRCC primary lesion with sarcomatoid differentiation

ccRCC—clear cell renal cell carcinoma, IVS—inferior vena cava, PET/CT—positron emission tomography/computed tomography, PSMA—prostate-specific membrane antigen, RCC—renal cell carcinoma.

**Table 4:** The detection rate of PSMA PET/CT in clinical studies evaluating its diagnostic accuracy in ccRCC.

Authors [Ref.]	Number of CcRCC Patient Included	Tracer	Comparative Imaging/Tests	Detection Rate (%)
Li et al. [52]	40	[^68^Ga]Ga-PSMA-11	CT and/or MRI, histopathology	93.6 ‘per patient’ analysis
Raveenthiran et al. [54]	28	[^68^Ga]Ga-PSMA-11	CT	80.5 ‘per lesion’ analysis
Udovicich et al. [55]	54	[^68^Ga]Ga-PSMA-11 [^18^F]F-DCFPyL	CT, [^18^F]FDG PET/CT	85 ‘per patient’ analysis
Tariq et al. [56]	14	[^18^F]F-PSMA-1007 [^68^Ga]Ga-PSMA-11	MRI	85.7 ‘per lesion’ analysis
Aggarwal et al. [60]	27	[^68^Ga]Ga-PSMA-11	CT, [^18^F]FDG PET/CT	95.2 ‘per lesion’ analysis
Gasparo et al. [61]	22	[^68^Ga]Ga-PSMA-HBED-CC	CT and/or MRI, histopathology	68 ‘per patient’ analysis
Rhee et al. [62]	8	[^68^Ga]Ga-PSMA-HBED-CC	CT, histopathology	92.1 * ‘per patient’ analysis
Sawicki et al. [63]	4	[^68^Ga]Ga-PSMA-HBED-CC	CT, histopathology	50 for pulmonary lesions
Meyer et al. [64]	14	[^18^F]F-DCFPyL	CT and/or MRI	92.8 ‘per patient’ analysis 88.9 ‘per lesion’ analysis
Liu et al. [65]	15	[^18^F]F-DCFPyL	[^18^F]FDG PET/CT	100 ‘per patient’ analysis
Rowe et al. [66]	5	[^18^F]F-DCFPyL	CT and/or MRI	94.7 ‘per lesion’ analysis
Tariq et al. [67]	10	[^18^F]F-PSMA-1007	[^18^F]FDG PET/CT	70 in ‘per patient’ analysis

* Data from a group of 10 RCC patients: 8 with ccRCC, 1 with pRCC and 1 with unclassified RCC. CT—computed tomography, MRI—magnetic resonance imaging, PET/CT—positron emission tomography/computed tomography, PSMA—prostate-specific membrane antigen.

**Table 5:** Visual sensitivity, specificity and SUVmax of kidney RCC and ccRCC lesions in individual studies with PSMA PET/CT. ccRCC, clear cell renal cell carcinoma; RCC, renal cell carcinoma.

Authors [Ref.]	Tracer	Number of RCC Kidney Lesions (Number of ccRCC Kidney Lesions)	Number of RCC (ccRCC) Patient Included	Visually Assessed Sensitivity (%) for RCC (ccRCC) Kidney Lesions	Visually Assessed Specificity (%) for RCC (ccRCC) Kidney Lesions	SUVmax for RCC and ccRCC Kidney Lesions
Tracers Excreted Mainly via Kidneys Route						
Golan et al. [46]	[^68^Ga]Ga-PSMA-11	29 RCC (unknown)	24 patients (18 ccRCC)	62% (unknown)	60% (unknown)	RCC: mean 10.6 ± 6.2; (range 1.5–22), median 10.6; IQR 5.4–15.8 ccRCC: unknown
Raveenthiran et al. [59]	[^68^Ga]Ga-PSMA-11	9 RCC (unknown)	9 patients (8 ccRCC)	77.8 (87.5%)	unknown (unknown)	unknown
Li et al. [51]	[^68^Ga]Ga-PSMA-11	16 RCC (9 ccRCC)	50 patients (40 ccRCC)	100% (100%)	100% (100%)	RCC: median 16.8 ccRCC: median 18.0
Aggarwal et al. [60]	[^68^Ga]Ga-PSMA-11	unknown (unknown)	37 patients (27 ccRCC)	unknown (unknown)	unknown (unknown)	RCC: median 16.2 IQR 10.7–23.6 ccRCC median 16.2 IQR 12.7–33.8
Gasparo et al. [61]	[^68^Ga]Ga-PSMA -HBED-CC	Unknown (unknown)	26 patients (22 ccRCC)	100% (100%)	unknown (unknown)	RCC: mean 19.3 (range 5.45–54.2) ccRCC: median 24.9 range 4.4–114.6
Rhee et al. [62]	[^68^Ga]Ga-PSMA -HBED-CC	unknown (unknown)	10 patients (8 ccRCC)	unknown (unknown)	unknown (unknown)	RCC: median 18.0 (3.7–36.5) ccRCC: unknown
Sawicki et al. [63]	[^68^Ga]Ga-PSMA HBED-CC	5 RCC (3 ccRCC)	6 patients (4 ccRCC)	unknown (unknown)	unknown (unknown)	RCC: mean 9.9 ± 9.2 range 1.7–27.2 ccRCC mean 13.5 ± 12.8; range 1.7–27.2
Tracers Excreted Mainly via Hepatic Route						
Meyer et al. [64]	[^18^F]F-DCFPyl	3 RCC (3 ccRCC)	14 patients (14 ccRCC)	100% (unknown)	-	ccRCC: median 9.6 (range 7.3–15.8)
Tariq et al. [67]	[^18^F]F-PSMA-1007	5 RCC (4 ccRCC)	11 patients (10 ccRCC)	80% (100%)	unknown (unknown)	RCC: range 3.2–13.7 ccRCC: unknown

**Table 6:** SUVmax of ccRCC lesions in individual studies with PSMA PET/CT.

Authors [Ref.]	Tracer	Main Excretion Route	SUVmax of All CcRCC Lesions	Primary CcRCC Lesion SUVmax	Metastatic ccRCC Lesions SUVmax
Tracers Excreted Mainly via Renal Route (or Both)					
Gasparo et al. [61]	[^68^Ga]Ga-PSMA-HBED-CC	renal	-	median 24.9; range 4.4–114.6 mean 34.1	range 3.2–66.6
Li et al. [52]	[^68^Ga]Ga-PSMA-11	renal	9	median 18.0	range 7.4–9.6
Ghune et al. [53]	[^68^Ga]Ga-PSMA-11	renal	9	-	median 3.1 (range 1.2–23.4)
Udovicich et al. [55]	[^68^Ga]Ga-PSMA-11, [^18^F]F-DCFPyL	Renal/hepatic	-	-	median 16.0
Rhee et al. [62]	[^68^Ga]Ga-PSMA-HBED-CC	renal	8	median 18.0 (3.7–36.5) * median 28.6 ^#^	mean 19.5 (range 1.5–48.0)
Tariq et al. [56]	[^18^F]F-PSMA-1007 [^68^Ga]Ga-PSMA-11	hepatic/renal	4.1–62.4	mean 25.3 range 6.1–62.4	range 4.1–59.9 (IVC tumor thrombi)
Sawicki et al. [63]	[^68^Ga]Ga-PSMA-HBED-CC	renal	4	mean 13.5 ± 12.8 range 1.7–27.2	mean 9.9 ± 8.3 (range 3.4–25.6)
Aggarwal et al. [60]	[^68^Ga]Ga-PSMA-11	renal	median 6.9 IQR 4.2–12.1	median 16.2 IQR 12.7–33.8	median 3.9–20.1 IQR 3.2–42.6
Tracers Excreted Mainly via Hepatic Route					
Meyer et al. [64]	[^18^F]F-DCFPyL	prospective	-	median 9.6 range 7.3–15.8	median 2.7 (range 0.9–38.5)
Liu et al. [65]	[^18^F]F-DCFPyL	retrospective	-	-	mean 6.9–soft tissue lesions mean 8.2–bone lesions
Rowe et al. [66]	[^18^F]F-DCFPyL	prospective	-	-	range 1.9–19.3

* data for a group of 10 RCC patients: 8 with ccRCC, 1 with pRCC and 1 with unclassified RCC; # case of ccRCC with sarcomatoid differentiation. ccRCC—clear cell renal cell carcinoma, IVS—inferior vena cava, RCC—renal cell carcinoma.

**Table 7:** SUVmax parameters, detection rates and specificity for deferent RCC metastases’ localization in PSMA PET/CT studies.

Location of Metastases	SUVmax	Detection Rate (%)	Specificity (%)
Liver	Mean 29.8 (range 11.2–48.3) [61] Median 20.1 (IQR 13.0–42.6) [60]	unknown	unknown
Lymph nodes	Mean 20.6 (range 7.6–35.4) [61] Median 9.6 [52] 1.2 (one lesion) [53] Range 2.2–8.2 [64] Median 11.6 (IQR 6.3–22.1) [60]	92.8 [43]	98.9 [43]
Bones	Mean 24.0 (range 10.0–46.3) [61] Median 7.5 [52] 23.4 (one lesion) [53] Mean 8.2 [54] Range 1.8–3.9 [64] Median 8.2 (IQR 5.0–11.0) [60]	100 [43]	90,4 [43]
Lung	Median 9.6 [52] Range 0.5–3.1 [53] Range 0.9–38.5 [64] Mean 12.08 (range 3.2–24.9) [59] Median 5.1 (IQR 3.5–9.0) [60]	71.4 [43] 50.0 [57]	100 [43]
Brain	Mean 15.5 (range: 10.7–20.2) [61] 2.5 (one lesion) [64] 3.9 (one lesion) [66] Median 3.9 (IQR 3.2–4.7) [60]	unknown	unknown

**Table 8:** Comparison of PSMA PET/CT and [^18^F]FDG PET/CT studies.

Authors [Ref.]	Number of CcRCC Patient Included	Tracer	Results
Udovicich et al. [55]	54	[^68^Ga]Ga-PSMA-11/ [^18^F]F-DCFPyL vs. [^18^F]FDG PET/CT	- most of s lesions showed uptake in both studies - lower uptake values on [18F]FDG PET/CT - in 5% of patients uptake only on [^18^F]FDG PET/CT
Tariq et al. [67]	10	[^18^F]F-PSMA-1007 vs. [^18^F]FDG PET/CT	- uptake of both tracers seen in 40% of cases, PSMA uptake alone in 20% [^18^F]FDG uptake alone in 40% of lesions
Liu et al. [65]	15	[^18^F]F-DCFPyL vs. [^18^F]FDG PET/CT	- PSMA PET/CT detected more metastatic ccRCC lesions within soft tissues, - higher detection rate of PSMA PET/CT in local recurrence of RCC and bone metastases
Aggarwal et al. [60]	27	[^68^Ga]Ga-PSMA-11 vs. [^18^F]FDG PET/CT	- PSMA PET/CT showed more lesions than [^18^F]FDG PET/CT, - higher uptake parameters in PSMA PET/CT

## Data Availability

No new data were created or analyzed in this study. Data sharing is not applicable to this article.

## Abbreviations

AML	angiomyolipoma
AUC	area under the curve
CAIX	carbonic anhydrase IX
ccRCC	clear cell renal cell carcinoma
chrRCC	chromophobe renal cell carcinoma
CNB	core-needle biopsies
CT	computed tomography
CTLA-4	cytotoxic T-lymphocyte antigen 4
EB	Evans blue
ECs	endothelial cells
FAPI	fibroblast activation protein inhibitor
[^18^F]FDG	[^18^F]-2-fluoro-2-deoxyglucose
GLUT-1	fructose 1,6-bisphosphatase 1
HIF-2α	hypoxia-inducible factor 2 alpha
HR	hazard ratio
IHC	immunohistochemistry
IVS	inferior vena cava
mCRPC	metastatic castration-resistant prostate cancer
MRI	magnetic resonance imaging
NCT	National Clinical Trial number
NM	nuclear medicine
OS	overall survival
PD-1	programmed cell death-1
PDGFR-β	platelet-derived growth factor-β
PD-1L	programmed cell death-1 ligand
PET/CT	positron emission tomography/computed tomography
PFS	progression-free survival
PPV	positive predictive value
pRCC	papillary renal cell carcinoma
PRISMA	Systematic Reviews and Meta-Analyses
PSMA	prostate-specific membrane antigen
PSMA-TV	PSMA-derived Tumor Volume
QUADAS-2	The Quality Assessment of Diagnostic Accuracy Studies
RCC	renal cell carcinoma
RGD	cyclic arginine-glycine-aspartate
RLT	radioligand therapy
SSTR	somatostatin receptors
SUVmax	maximal standard uptake value
SUVmean	mean standard uptake value
SUVpeak	peak standard uptake value
TBR	Tumor-to-Background Ratio
[^99m^Tc]Tc-EC	[^99m^Tc]Tc ethylenecysteine
[^99m^Tc]Tc-DMSA	[^99m^Tc]Tc-dimercaptosuccinic acid
[^99m^Tc]Tc-DTPA	[^99m^Tc]Tc-diethylenetriamine pentaacetic acid
[^99m^Tc]Tc-MAG3	[^99m^Tc]Tc-mercaptoacetyltriglycine
[^99m^Tc]Tc-MIBI	[^99m^Tc]Tc methoxyisobutyl isonitrile
[^99m^Tc]Tc-MDP	[^99m^Tc]Tc methylene diphosphate
TKI	tyrosine kinase inhibitor
TL-PSMA	Total Lesion PSMA
US	ultrasound
VEGF	vascular endothelial growth factor
VEGFR-2	vascular endothelial growth factor receptor-2

## References

[1] Chow W.H., Dong L.M., Devesa S.S. (2010). Epidemiology and risk factors for kidney cancer. Nat. Rev. Urol..

[2] Thorstenson A., Bergman M., Scherman-Plogell A.H., Hosseinnia S., Ljungberg B., Adolfsson J., Lundstam S. (2014). Tumor characteristics and surgical treatment of renal cell carcinoma in Sweden 2005–2010: A population-based study from the national Swedish kidney cancer register. Scand. J. Urol..

[3] Ferlay J., Colombet M., Soerjomataram I., Dyba T., Randi G., Bettio M., Gavin A., Visser O., Bray F. (2018). Cancer incidence and mortality patterns in Europe: Estimates for 40 countries and 25 major cancers in 2018. Eur. J. Cancer.

[4] Bray F., Ferlay J., Soerjomataram I., Siegel R.L., Torre L.A., Jemal A. (2018). Global cancer statistics 2018: GLOBOCAN estimates of incidence and mortality worldwide for 36 cancers in 185 countries. CA Cancer J. Clin..

[5] Alongi P., Picchio M., Zattoni F., Spallino M., Gianolli L., Saladini G., Evangelista L. (2016). Recurrent renal cell carcinoma: Clinical and prognostic value of FDG PET/CT. Eur. J. Nucl. Med. Mol. Imaging.

[6] Inamura K. (2017). Renal cell tumors: Understanding their molecular pathological epidemiology and the 2016 del classification. Int. J. Mol. Sci..

[7] Powles T., Albiges L., Bex A., Comperat E., Grünwald V., Kanesvaran R., Kitamura H., McKay R., Porta C., Procopio G. (2024). Renal cell carcinoma: ESMO Clinical Practice Guideline for diagnosis, treatment and follow-up. Ann. Oncol..

[8] Marconi L., Dabestani S., Lam T.B., Hofmann F., Stewart F., Norrie J., Bex A., Bensalah K., Canfield S.E., Hora M. (2016). Systematic Review and Meta-analysis of Diagnostic Accuracy of Percutaneous Renal Tumour Biopsy. Eur. Urol..

[9] Kane C.J., Mallin K., Ritchey J., Cooperberg M.R., Carroll P.R. (2008). Renal cell cancer stage migration: Analysis of the National Cancer Data Base. Cancer.

[10] Azawi N.H., Tesfalem H., Mosholt K.S., Høyerup P., Jensen E.S., Malchau E., Fode M. (2016). Recurrence rates and survival in a Danish cohort with renal cell carcinoma. Dan. Med. J..

[11] Leibovich B.C., Blute M.L., Cheville J.C., Lohse C.M., Frank I., Kwon E.D., Weaver A.L., Parker A.S., Zincke H. (2003). Prediction of progression after radical nephrectomy for patients with clear cell renal cell carcinoma: A stratification tool for prospective clinical trials. Cancer.

[12] Gupta K., Miller J.D., Li J.Z., Russell M.W., Charbonneau C. (2008). Epidemiologic and socioeconomic burden of metastatic renal cell carcinoma (mRCC): A literature review. Cancer Treat. Rev..

[13] Motzer R.J., Jonasch E., Agarwal N., Alva A., Baine M., Beckermann K., Carlo M.I., Choueiri T.K., Costello B.A., Derweesh I.H. (2022). Kidney Cancer, Version 3.2022, NCCN Clinical Practice Guidelines in Oncology. J. Natl. Compr. Cancer Netw..

[14] Ljungberg B., Albiges L., Abu-Ghanem Y., Bedke J., Capitanio U., Dabestani S., Fernández-Pello S., Giles R.H., Hofmann F., Hora M. (2022). European Association of Urology Guidelines on Renal Cell Carcinoma: The 2022 Update. Eur. Urol..

[15] Mettler M., Guiberteau M.J. (2019). Genitourinary system and adrenal glands. Essentials of Nuclear Medicine.

[16] Kabasakal L., Turoğlu H.T., Onsel C., Ozker K., Uslu I., Atay S., Cansiz T., Sönmezoğlu K., Altiok E., Isitman A.T. (1995). Clinical comparison of technetium-99m-EC, technetium-99m-MAG3 and iodine-131-OIH in renal disorders. J. Nucl. Med..

[17] Blaufox M.D., De Palma D., Taylor A., Szabo Z., Prigent A., Samal M., Li Y., Santos A., Testanera G., Tulchinsky M. (2018). The SNMMI and EANM practice guideline for renal scintigraphy in adults. Eur. J. Nucl. Med. Mol. Imaging.

[18] Vitti R.A., Maurer A.H. (1985). Single photon emission computed tomography and renal pseudotumor. Clin. Nucl. Med..

[19] Rowe S.P., Gorin M.A., Gordetsky J., Ball M.W., Pierorazio P.M., Higuchi T., Epstein J.I., Allaf M.E., Javadi M.S. (2015). Initial experience using 99mTc-MIBI SPECT/CT for the differentiation of oncocytoma from renal cell carcinoma. Clin. Nucl. Med..

[20] Tataru O.S., Marchioni M., Crocetto F., Barone B., Lucarelli G., Del Giudice F., Busetto G.M., Veccia A., Lo Giudice A., Russo G.I. (2023). Molecular Imaging Diagnosis of Renal Cancer Using 99mTc-Sestamibi SPECT/CT and Girentuximab PET-CT-Current Evidence and Future Development of Novel Techniques. Diagnostics.

[21] Sistani G., Bjazevic J., Kassam Z., Romsa J., Pautler S. (2021). The value of (99m)Tc-sestamibi single-photon emission computed tomography-computed tomography in the evaluation and risk stratification of renal masses. Can. Urol. Assoc. J..

[22] Sohaib S.A., Cook G., Allen S.D., Hughes M., Eisen T., Gore M. (2009). Comparison of whole-body MRI and bone scintigraphy in the detection of bone metastases in renal cancer. Br. J. Radiol..

[23] Aide N., Cappele O., Bottet P., Bensadoun H., Regeasse A., Comoz F., Sobrio F., Bouvard G., Agostini D. (2003). Effiency of [18F]FDG PET in characterizing renal cancer and detecting distant metastases: A comparison with CT. Eur. J. Nucl. Med. Mol. Imaging.

[24] Chen R., Zhou X., Huang G., Liu J. (2019). Bisphosphatase 1 expression reduces 18 F-FDG uptake in clear cell Renal Cell Carcinoma. Contrast Media Mol. Imaging.

[25] Wang H.Y., Ding H.J., Chen J.H., Chao C.H., Lu Y.Y., Lin W.Y., Kao C.H. (2012). Meta-analysis of the diagnostic performance of [18F]FDG-PET and PET/CT in renal cell carcinoma. Cancer Imaging.

[26] Ma H., Shen G., Liu B., Yang Y., Ren P., Kuang A. (2017). Diagnostic performance of 18F-FDG PET or PET/CT in restaging renal cell carcinoma: A systematic review and meta-analysis. Nucl. Med. Commun..

[27] Ueno D., Yao M., Tateishi U., Minamimoto R., Makiyama K., Hayashi N., Sano F., Murakami T., Kishida T., Miura T. (2012). Early assessment by FDG-PET/CT of patients with advanced renal cell carcinoma treated with tyrosine kinase inhibitors is predictive of disease course. BMC Cancer.

[28] Nakaigawa N., Kondo K., Kaneta T., Tateishi U., Minamimoto R., Namura K., Ueno D., Kobayashi K., Kishida T., Ikeda I. (2018). FDG PET/CT after first molecular targeted therapy predicts survival of patients with renal cell carcinoma. Cancer Chemother. Pharmacol..

[29] Kayani I., Avril N., Bomanji J., Chowdhury S., Rockall A., Sahdev A., Nathan P., Wilson P., Shamash J., Sharpe K. (2011). Sequential FDG-PET/CT as a biomarker of response to Sunitinib in metastatic clear cell renal cancer. Clin. Cancer Res..

[30] Chen J.L., Appelbaum D.E., Kocherginsky M., Cowey C.L., Rathmell W.K., McDermott D.F., Stadler W.M. (2013). FDG-PET as a predictive biomarker for therapy with everolimus in metastatic renal cell cancer. Cancer Med..

[31] Civan C., Kuyumcu S., Has Simsek D., Sanli O., Isik E.G., Ozkan Z.G., Hurdogan O., Sanli Y. (2024). The role of [68 Ga]Ga-FAPI-04 PET/CT in renal cell carcinoma: A preliminary study. Eur. J. Nucl. Med. Mol. Imaging.

[32] Yin Q., Xu H., Zhong Y., Ni J., Hu S. (2022). Diagnostic performance of MRI, SPECT, and PET in detecting renal cell carcinoma: A systematic review and meta-analysis. BMC Cancer.

[33] Nakamoto Y., Ishimori T., Shimizu Y., Sano K., Togashi K. (2019). Clinical utility of ^68^Ga-DOTATOC positron emission tomography/computed tomography for recurrent renal cell carcinoma. Eur. J. Nucl. Med. Mol. Imaging.

[34] Oyama N., Ito H., Takahara N., Miwa Y., Akino H., Kudo T., Okazawa H., Fujibayashi Y., Komatsu K., Tsukahara K. (2014). Diagnosis of complex renal cystic masses and solid renal lesions using PET imaging: Comparison of 11C-acetate and 18F-FDG PET imaging. Clin. Nucl. Med..

[35] Sharma P., Karunanithi S., Chakraborty P.S., Kumar R., Seth A., Julka P.K., Bal C., Kumar R. (2014). 18F-fuoride PET/CT for detection of bone metastasis in patients with renal cell carcinoma: A pilot study. Nucl. Med. Commun..

[36] Guo C., Liu Y., Yang H., Xia Y., Li X., Chen L., Feng Y., Zhang Y., Chen Y., Huang Z. (2024). A pilot study of [68Ga]Ga-fibroblast activation protein inhibitor-04 PET/CT in renal cell carcinoma. Br. J. Radiol..

[37] Fuccio C., Spinapolice E.G., Cavalli C., Palumbo R., D’Ambrosio D., Trifirò G. (2013). 18F-Fluoride PET/CT in the detection of bone metastases in clear cell renal cell carcinoma: Discordance with bone scintigraphy. Eur. J. Nucl. Med. Mol. Imaging.

[38] Smaldone M.C., Chen D.Y., Yu J.Q., Plimack E.R. (2012). Potential role of (124)I-girentuximab in the presurgical diagnosis of clear-cell renal cell cancer. Biologics.

[39] Lin R., Wang C., Chen S., Lin T., Cai H., Chen S., Yang Y., Zhang J., Xu F., Zhang J. (2024). [68Ga]Ga-LNC1007 PET/CT in the evaluation of renal cell carcinoma: Comparison with 2-[18F]FDG/[68Ga]Ga-PSMA PET/CT. Eur. J. Nucl. Med. Mol. Imaging.

[40] Zhang J., Lu T., Lu S., Ma S., Han D., Zhang K., Xu C., Liu S., Gan L., Wu X. (2023). Single-cell analysis of multiple cancer types reveals differences in endothelial cells between tumors and normal tissues. Comput. Struct. Biotechnol. J..

[41] Pełka K., Bodys-Pełka A., Kunikowska J. (2023). Prostate-specific membrane antigen expression in intracranial lesions—A review of the primary, metastatic, and nonneoplastic lesions. Nucl. Med. Rev. Cent. East. Eur..

[42] Rogic I., Golubic A.T., Zuvic M., Smitran T., Jukic N., Gamulin M., Kastelan Z., Huic D. (2024). Clinical utility of [68Ga]Ga-PSMA-11 PET/CT in initial staging of patients with prostate cancer and importance of intraprostatic SUVmax values. Nucl. Med. Rev. Cent. East. Eur..

[43] Heng D.Y., Xie W., Regan M.M., Warren M.A., Golshayan A.R., Sahi C., Eigl B.J., Ruether J.D., Cheng T., North S. (2009). Prognostic Factors for Overall Survival in Patients with Metastatic Renal Cell Carcinoma Treated with Vascular Endothelial Growth Factor–Targeted Agents: Results from a Large, Multicenter Study. J. Clin. Oncol..

[44] Baccala A., Sercia L., Li J., Heston W., Zhou M. (2007). Expression of prostate specific membrane antigen in tumor-associated neovasculature of renal neoplasms. Urology.

[45] Campbell S.P., Baras A.S., Ball M.W., Kates M., Hahn N.M., Bivalacqua T.J., Johnson M.H., Pomper M.G., Allaf M.E., Rowe S.P. (2018). Low levels of PSMA expression limit the utility of 18F-DCFPyL PET/CT for imaging urothelial carcinoma. Ann. Nucl. Med..

[46] Golan S., Aviv T., Groshar D., Yakimov M., Zohar Y., Prokocimer Y., Nadu A., Baniel J., Domachevsky L., Bernstine H. (2021). Dynamic ^68^Ga-PSMA-11 PET/CT for the Primary Evaluation of Localized Renal Mass: A Prospective Study. J. Nucl. Med..

[47] Chang S.S., Reuter V.E., Heston W.D., Gaudin P.B. (2001). Metastatic renal cell carcinoma neovasculature expresses prostate-specific membrane antigen. Urology.

[48] Li G., Lambert C., Gentil-Perret A., Genin C., Tostain J. (2003). Molecular and cytometric analysis of renal cell carcinoma cells. Concepts, techniques and prospects. Prog. Urol..

[49] Al-Ahmadie H.A., Olgac S., Gregor P.D., Tickoo S.K., Fine S.W., Kondagunta G.V., Scher H.I., Morris M.J., Russo P., Motzer R.J. (2008). Expression of prostate-specific membrane antigen in renal cortical tumors. Mod. Pathol..

[50] Spatz S., Tolkach Y., Jung K., Stephan C., Busch J., Ralla B., Rabien A., Feldmann G., Brossart P., Bundschuh R.A. (2018). Comprehensive evaluation of prostate specific membrane antigen expression in the vasculature of renal tumors: Implications for imaging studies and prognostic role. J. Urol..

[51] Demirci E., Ocak M., Kabasakal L., Decristoforo C., Talat Z., Halaç M., Kanmaz B. (2014). ^68^Ga-PSMA PET/CT imaging of metastatic clear cell renal cell carcinoma. Eur. J. Nucl. Med. Mol. Imaging.

[52] Li Y., Zheng R., Zhang Y., Huang C., Tian L., Liu R., Liu Y., Zhang Z., Han H., Zhou F. (2022). Special issue “The advance of solid tumor research in China”: ^68^Ga-PSMA-11 PET/CT for evaluating primary and metastatic lesions in different histological subtypes of renal cell carcinoma. Int. J. Cancer.

[53] Gühne F., Seifert P., Theis B., Steinert M., Freesmeyer M., Drescher R. (2021). PSMA-PET/CT in Patients with Recurrent Clear Cell Renal Cell Carcinoma: Histopathological Correlations of Imaging Findings. Diagnostics.

[54] Gao J., Xu Q., Fu Y., He K., Zhang C., Zhang Q., Shi J., Zhao X., Wang F., Guo H. (2021). Comprehensive evaluation of ^68^Ga-PSMA11 PET/CT parameters for discriminating pathological characteristics in primary clear-cell renal cell carcinoma. Eur. J. Nucl. Med. Mol. Imaging.

[55] Udovicich C., Callahan J., Bressel M., Ong W.L., Perera M., Tran B., Azad A., Haran S., Moon D., Chander S. (2022). Impact of Prostate-specific Membrane Antigen Positron Emission Tomography/Computed Tomography in the Management of Oligometastatic Renal Cell Carcinoma. Eur. Urol. Open Sci..

[56] Tariq A., McGeorge S., Pearce A., Rhee H., Wood S., Kyle S., Marsh P., Raveenthiran S., Wong D., McBean R. (2022). Characterization of tumor thrombus in renal cell carcinoma with prostate specific membrane antigen (PSMA) positron emission tomography (PET)/computed tomography (CT). Urol. Oncol. Semin. Orig. Investig..

[57] Gao J., Meng L., Xu Q., Zhao X., Deng Y., Fu Y., Guo S., He K., Shi J., Wang F. (2022). ^68^Ga-PSMA-11 PET/CT Parameter Correlates with Pathological VEGFR-2/PDGFR-β Expression in Renal Cell Carcinoma Patients. Mol. Imaging Biol..

[58] Meng L., Zhang S., Gao J., Xu Q., Fu Y., Zhou Y.H., Wang F., Guo H. (2022). [68Ga]Ga-PSMA-11 PET/CT has potential application in predicting tumor HIF-2α expression and therapeutic response to HIF-2α antagonists in patients with RCC. Eur. Radiol..

[59] Raveenthiran S., Esler R., Yaxley J., Kyle S. (2019). The use of 68Ga-PET/CT PSMA in the staging of primary and suspected recurrent renal cell carcinoma. Eur. J. Nucl. Med. Mol. Imaging.

[60] Aggarwal P., Singh H., Das C.K., Mavuduru R.S., Kakkar N., Lal A., Gorsi U., Kumar R., Mittal B.R. (2024). Potential role of ^68^Ga-PSMA PET/CT in metastatic renal cell cancer: A prospective study. Eur. J. Radiol..

[61] Gasparro D., Scarlattei M., Silini E.M., Migliari S., Baldari G., Cervati V., Graziani T., Campanini N., Maestroni U., Ruffini L. (2023). High Prognostic Value of ^68^Ga-PSMA PET/CT in Renal Cell Carcinoma and Association with PSMA Expression Assessed by Immunohistochemistry. Diagnostics.

[62] Rhee H., Blazak J., Tham C.M., Ng K.L., Shepherd B., Lawson M., Preston J., Vela I., Thomas P., Wood S. (2016). Pilot study: Use of gallium-68 PSMA PET for detection of metastatic lesions in patients with renal tumour. EJNMMI Res..

[63] Evangelista L., Basso U., Maruzzo M., Novara G. (2020). The role of radiolabeled prostate-specific membrane antigen positron emission tomography/computed tomography for the evaluation of renal cancer. Eur. Urol. Focus.

[64] Meyer A.R., Carducci M.A., Denmeade S.R., Markowski M.C., Pomper M.G., Pierorazio P.M., Allaf M.E., Rowe S.P., Gorin M.A. (2019). Improved identification of patients with oligometastatic clear cell renal cell carcinoma with PSMA-targeted 18F-DCFPyL PET/CT. Ann. Nucl. Med..

[65] Liu Y., Wang G., Yu H., Wu Y., Lin M., Gao J., Xu B. (2020). Comparison of 18F-DCFPyL and 18F-FDG PET/computed tomography for the restaging of clear cell renal cell carcinoma: Preliminary results of 15 patients. Nucl. Med. Commun..

[66] Rowe S.P., Gorin M.A., Hammers H.J., Som Javadi M., Hawasli H., Szabo Z., Cho S.Y., Pomper M.G., Allaf M.E. (2015). Imaging of metastatic clear cell renal cell carcinoma with PSMA-targeted F-DCFPyL PET/CT. Ann. Nucl. Med..

[67] Tariq A., Kwok M., Pearce A., Rhee H., Kyle S., Marsh P., Raveenthiran S., Wong D., McBean R., Westera J. (2022). The role of dual tracer PSMA and FDG PET/CT in renal cell carcinoma (RCC) compared to conventional imaging: A multi-institutional case series with intra-individual comparison. Urol. Oncol. Semin. Orig. Investig..

[68] Urso L., Castello A., Rocca G.C., Lancia F., Panareo S., Cittanti C., Uccelli L., Florimonte L., Castellani M., Ippolito C. (2022). Role of PSMA-ligands imaging in Renal Cell Carcinoma management: Current status and future perspectives. J. Cancer Res. Clin. Oncol..

[69] Rizzo A., Racca M., Dall’Armellina S., Rescigno P., Banna G.L., Albano D., Dondi F., Bertagna F., Annunziata S., Treglia G. (2023). The Emerging Role of PET/CT with PSMA-Targeting Radiopharmaceuticals in Clear Cell Renal Cancer: An Updated Systematic Review. Cancers.

[70] Singhal T., Singh P., Parida G.K., Rescigno P., Banna G.L., Albano D., Dondi F., Bertagna F., Annunziata S., Treglia G. (2024). Role of PSMA-targeted PET-CT in renal cell carcinoma: A systematic review and meta-analysis. Ann. Nucl. Med..

[71] Sartor O., de Bono J., Chi K.N., Fizazi K., Herrmann K., Rahbar K., Tagawa S.T., Nordquist L.T., Vaishampayan N., El-Haddad G.l. (2021). Lutetium-177-PSMA-617 for metastatic castration-resistant prostate cancer. N. Engl. J. Med..

[72] Hofman M.S., Emmett L., Sandhu S., Iravani A., Joshua A.M., Goh J.C., Pattison D.A., Tan T.H., Kirkwood I.D., Ng S. (2021). ^177^Lu-PSMA-617 versus cabazitaxel in metastatic castration-resistant prostate cancer (TheraP): A randomised, open-label, phase 2 trial. Lancet.

[73] Calais J., Czernin J., Thin P., Gartmann J., Nguyen K., Armstrong W.R., Allen-Auerbach M., Quon A., Bahri S., Gupta P. (2021). Safety of PSMA-targeted molecular radioligand therapy with ^177^Lu-PSMA-617: Results from the prospective multicenter phase 2 trial RESIST-PC (NCT03042312). J. Nucl. Med..

[74] de Vries L.H., Lodewijk L., Braat A., Krijger G.C., Valk G.D., Lam M.G.E.H., Borel Rinkes I.H.M., Vriens M.R., de Keizer B. (2020). ^68^Ga-PSMA PET/CT in radioactive iodine-refractory differentiated thyroid cancer and first treatment results with ^177^Lu-PSMA-617. EJNMMI Res..

[75] Kunikowska J., Charzynska I., Kulinski R., Pawlak D., Maurin M., Królicki L. (2020). Tumor uptake in glioblastoma multiforme after IV injection of [^177^Lu]Lu-PSMA-617. Eur. J. Nucl. Med. Mol. Imaging.

[76] Klein Nulent T.J.W., van Es R.J.J., Krijger G.C., de Bree R., Willems S.M., de Keizer B. (2017). Prostate-specific membrane antigen PET imaging and immunohistochemistry in adenoid cystic carcinoma-a preliminary analysis. Eur. J. Nucl. Med. Mol. Imaging.

[77] Hirmas N., Leyh C., Sraieb M., Barbato F., Schaarschmidt B.M., Umutlu L., Nader M., Wedemeyer H., Ferdinandus J., Rischpler C. (2021). [^68^Ga]Ga-PSMA-11 PET/CT improves tumor detection and impacts management in patients with hepatocellular carcinoma (HCC). J. Nucl. Med..

[78] Zhang J., Schuchardt C., Chen X., Baum R.P. (2023). Rapid Tumor Washout of ^177^Lu-PSMA Radioligand in Renal Cell Carcinoma. Clin. Nucl. Med..

[79] Zang J., Fan X., Wang H., Liu Q., Wang J., Li H., Li F., Jacobson O., Niu G., Zhu Z. (2019). First-in-human study of ^177^Lu-EB-PSMA-617 in patients with metastatic castration-resistant prostate cancer. Eur. J. Nucl. Med. Mol. Imaging.

[80] Zang J., Liu Q., Sui H., Wang R., Jacobson O., Fan X., Zhu Z., Chen X. (2020). ^177^Lu-EB-PSMA Radioligand Therapy with Escalating Doses in Patients with Metastatic Castration-Resistant Prostate Cancer. J. Nucl. Med..

[81] Divgi C.R., Uzzo R.G., Gatsonis C., Bartz R., Treutner S., Yu J.Q., Chen D., Carrasquillo J.A., Larson S., Bevan P. (2013). Positron emission tomography/computed tomography identification of clear cell renal cell carcinoma: Results from the REDECT trial. J. Clin. Oncol..

[82] Shuch B., Pantuck A.J., Bernhard J.C., Morris M.A., Master V., Scott A.M., van Praet C., Bailly C., Önal B., Aksoy T. (2024). [89Zr]Zr-girentuximab for PET-CT imaging of clear-cell renal cell carcinoma: A prospective, open-label, multicentre, phase 3 trial. Clin. Trial. Lancet Oncol..

[83] Nakaigawa N., Hasumi H., Utsunomiya D., Yoshida K., Ishiwata Y., Oka T., Hayward C., Makiyama K. (2024). Evaluation of PET/CT imaging with [^89^Zr]Zr-DFO-girentuximab: A phase 1 clinical study in Japanese patients with renal cell carcinoma (Zirdac-JP). Jpn. J. Clin. Oncol..

[84] van Oostenbrugge T.J., Langenhuijsen J.F., Oosterwijk E., Boerman O.C., Jenniskens S.F., Oyen W.J.G., Fütterer J.J., Mulders P.F.A. (2020). Follow-up imaging after cryoablation of clear cell renal cell carcinoma is feasible using single photon emission computed tomography with 111In-girentuximab. Eur. J. Nucl. Med. Mol. Imaging.

[85] Luna-Gutiérrez M., Cruz-Nova P., Jiménez-Mancilla N., Oros-Pantoja R., Lara-Almazán N., Santos-Cuevas C., Azorín-Vega E., Ocampo-García B., Ferro-Flores G. (2023). Synthesis and Evaluation of ^177^Lu-DOTA-PD-L1-i and 225Ac-HEHA-PD-L1-i as Potential Radiopharmaceuticals for Tumor Microenvironment-Targeted Radiotherapy. Int. J. Mol. Sci..

